# Special issue of BMC medical informatics and decision making on health natural language processing

**DOI:** 10.1186/s12911-019-0777-0

**Published:** 2019-04-04

**Authors:** V. G. Vinod Vydiswaran, Yaoyun Zhang, Yanshan Wang, Hua Xu

**Affiliations:** 1 Department of Learning Health Sciences, University of Michigan, Ann Arbor, MI USA; 2 School of Biomedical Informatics, The University of Texas Health Science Center at Houston, Houston, TX USA; 3 Department of Health Sciences Research, Mayo Clinic, Rochester, MN USA

Natural Language Processing (NLP) technologies have received significant attention in the medical domain and have demonstrated numerous successful uses in healthcare applications. The first international workshop on Health Natural Language Processing (HealthNLP 2018) provided a dedicated platform for close interactions among scholars, students, and industry professionals who are interested in NLP over health data such as clinical notes, social media, and biomedical literature. The workshop was organized in conjunction with IEEE International Conference on Healthcare Informatics (ICHI), and attracted submissions in the form of research papers, poster abstracts, and demonstration papers. All submissions were subjected to rigorous peer-review, with at least two peer reviews and at least one review by a senior member of the program committee. Selected papers and abstracts were featured as oral / poster presentations at the workshop. High quality research submissions were invited to expand their submitted works for a special issue of BMC Medical Informatics and Decision Making. The special issue on Health Natural Language Processing consists of twelve high quality papers, focusing on novel methods and applications of NLP over heath data. In the rest of this editorial, we highlight the key problems, approaches, and results from the papers included in this issue.

One of the major themes emerging from the papers is to develop novel machine learning algorithms and NLP approaches to process clinical notes for specific tasks. Three papers propose deep learning models for clinical text classification, parsing, and information extraction tasks. Yao et al. [1] proposed a clinical text classification method that combines rule-based features and knowledge-guided deep-learning techniques. Trigger phrases were identified using rules and used to label a seed example set, which was then used to train a convolutional neural network (CNN). The authors demonstrated that CNN models are effective in capturing domain knowledge and learning hidden features using word and CUI embeddings. Their proposed model outperformed state-of-the-art methods in the i2b2 Obesity challenge [2]. Zhang et al. [3] conducted a systematic comparison of deep learning based dependency parsers for the medical domain. They investigated four state-of-the-art deep learning based dependency parsers over two Treebank corpora – the MiPACQ Treebank and a Treebank of progress notes. The authors found that while retraining on medical corpora helps in general, the transition-based parsers demonstrated stronger generalizability on different treebanks than the graph-based parsers. Tang et al. [4] proposed a deep neural network, called attention-based CNN-LSTM-CRF, to capture local context information and select relevant words to recognize entities in Chinese clinical text. They showed that CNNs and attention mechanism are individually beneficial to LSTM-CRF-based clinical entity recognition system, and that the contribution of the attention mechanism is greater than just the CNN model. In another work, Wang et al. [5] developed a rule-based NLP algorithm to identify skeletal site-specific fractures from radiology reports, and verified it on the radiology reports from a rural community-based cohort.

Another focus area for papers in this issue revolves around challenges introduced by inefficient recording and management of clinical metadata in EHR systems. Hanauer et al. [6] took a closer look at the variations and complexities in the way numbers and numerical concepts are mentioned in clinical notes and their impact on information extraction systems. Using number-based keyword queries on a clinical text search engine, the authors highlighted the data quality issues in clinical notes and potential impact on subsequent tasks such as cohort identification. Cook et al. [7] developed text-based approaches to maintain accurate provider directories based on the Healthcare Provider Taxonomy code, location, name, and address information to match the state and federal records. Wang et al. [8] investigated the associations between problem list and practice setting using NLP and topic modeling techniques. Their method generated prioritized and meaningful problem lists corresponding to specific practice settings. Kreuzthaler et al. [9] proposed a clustering approach to compress redundant problem lists in electronic health records and create semantic topic spaces.

Availability of shareable clinical corpora is critical for advancing clinical NLP research. Weng et al. [10] assessed the willingness to share clinical data among a large cohort of proxy-for-consented individuals from two sites. They found that a substantial fraction of consented patients would be willing to donate de-identified clinical data to a shared research repository, and that individuals were most reluctant in sharing mental health, substance abuse, and domestic violence data. Sharma et al. [11] proposed a phenotyping system that could facilitate portability across different institutions and data systems. The prototyping system integrates rule-based and statistical machine learning approaches to extract clinically relevant features from the unstructured text. Portability is achieved by storing components in OMOP CDM (Common Data Model) formats, thus enabling the reuse, adaptation, and extension of rule-based clinical NLP systems.

Finally, acknowledging the current trend of availability and popularity of health information online, there were two papers that studied challenges introduced by the advent of health-related social media as a mechanism for patients to communicate with each other. Vydiswaran and Reddy [12] introduced the notion of peer expertise in online health forums and studied approaches to identify peer experts based on the communication patterns in forums. They postulated that patients and other caregivers often take on the roles of experts in online forums and are willing to share their experience with other users. Identifying them in health forums can help better develop stronger communities and patient support groups. In another work, Doan et al. [13] studied the issue of causality extraction from Twitter messages. They develop lexico-syntactic patterns based on dependency parser outputs to extract health-related causal relations in three health-related topics. Manual analysis on extracted casualties in tweets revealed interesting insights into health-related discussion on Twitter.

In conclusion, these papers highlight the plethora of research in health-related natural language processing. While advancements are being made in meaningful use of electronic health records for clinical decision support, these papers represent a snapshot of the broad spectrum of challenges where natural language processing can help advance the methodologies, tools, and applications in healthcare.
